# Defects Contributing to Hysteresis in Few-Layer and Thin-Film MoS_2_ Memristive Devices

**DOI:** 10.3390/ma17061350

**Published:** 2024-03-15

**Authors:** Saadman Abedin, Vladislav Kurtash, Sobin Mathew, Sebastian Thiele, Heiko O. Jacobs, Jörg Pezoldt

**Affiliations:** FG-Nanotechnologie, Institut für Mikro-und Nanoelektronik, Institut für Mikro-und Nanotechnologien MacroNano®, Institut für Werkstofftechnik, TU Ilmenau, Postfach 100565, 98684 Ilmenau, Germany; saadman.abedin@tu-ilmenau.de (S.A.); sobin.mathew@tu-ilmenau.de (S.M.); thielesebastian@outlook.com (S.T.); heiko.jacobs@tu-ilmenau.de (H.O.J.)

**Keywords:** semiconductor memory devices, memristors, neuromorphic computing systems, charge-trapping devices, molybdenum disulfide

## Abstract

Molybdenum disulfide, a two-dimensional material extensively explored for potential applications in non-von Neumann computing technologies, has garnered significant attention owing to the observed hysteresis phenomena in MoS_2_ FETs. The dominant sources of hysteresis reported include charge trapping at the channel–dielectric interface and the adsorption/desorption of molecules. However, in MoS_2_ FETs with different channel thicknesses, the specific nature and density of defects contributing to hysteresis remain an intriguing aspect requiring further investigation. This study delves into memristive devices with back-gate modulated channel layers based on CVD-deposited flake-based and thin-film-based MoS_2_ FETs, with a few-layer (FL) and thin-film (TF) channel thickness. Analysis of current–voltage (I−V) and conductance–frequency ($G_{p}/\omega -f$) measurements led to the conclusion that the elevated hysteresis observed in TF MoS_2_ devices, as opposed to FL devices, stems from a substantial contribution from intrinsic defects within the channel volume, surpassing that of interface defects. This study underscores the significance of considering both intrinsic defects within the bulk and the interface defects of the channel when analyzing hysteresis in MoS_2_ FETs, particularly in TF FETs. The selection between FL and TF MoS_2_ devices depends on the requirements for memristive applications, considering factors such as hysteresis tolerance and scaling capabilities.

## 1. Introduction

For advanced technology nodes, molybdenum disulfide (MoS_2_), a widely studied two-dimensional (2D) material, has raised the expectations to accommodate further scaling of the channel length, mitigate short-channel effects, and enhance electrostatic gate control beyond what is achievable for Si-based devices. MoS_2_ as a channel layer in field-effect transistors (FETs) has demonstrated remarkable performance metrics, including high mobility ($\mu_{fe}∼$200–400 cm^2^/Vs), large on/off current ratio ($I_{on}/I_{off}∼$$10^{6}$ to $10^{8}$), and nearly ideal sub-threshold swing (SS∼65 mV/decade) [1,2,3,4]. It has been identified as a promising platform beyond Si for developing next-generation transistors operating at sub 5 nm regimes stemming from several advantageous factors [1,2,4,5,6,7]. Firstly, MoS_2_ is compatible with existing Si-based device technologies [5,8,9,10,11]. Secondly, it can be integrated into diverse heterostructures without being limited by lattice mismatch constraints [8,9,10,11,12]. Thirdly, it possesses a tunable bandgap well-suited for efficient electronic device switching [6,8,11,13]. As the layer thickness decreases, the bandgap of MoS_2_ widens and transitions from indirect to direct, ranging from ∼1.2 to 1.8 eV from bulk to monolayer [3,4,11,13,14,15,16,17]. Moreover, its atomic-layer precision provides excellent electrostatic gate control down to the monolayer limit and significantly reduces power dissipation in the off state [2,4,5,6,8,10,11]. Furthermore, its higher carrier effective mass (MoS_2_: $m_{n}^{*}$∼$0.55m_{0}$, Si [100]: $m_{n}^{*}$∼$0.19m_{0}$) along the transport direction helps reduce direct source-to-drain tunneling, and its lower in-plane dielectric constant (MoS_2_:∼4, Si:∼11.7) impedes drain field in highly scaled transistors [2,4,6,11]. These combined attributes position MoS_2_ as a strong candidate as a channel material in future transistor operation at sub 5 nm scaling limits, outperforming the capabilities of Si [1,2,4,6,18].

Recent years have witnessed a growing interest in FETs based on MoS_2_ nanosheets due to the hysteresis phenomena observed in their conductance, presenting opportunities to be utilized in non-von Neumann computing technologies [5,9,10,12,19,20]. However, understanding the nature of hysteresis in MoS_2_ FETs is complex and diverse, in particular due to the variations across devices and cycles [10,12,19,21]. Determining and distinguishing the factors contributing to hysteresis and comprehending their associated behavior in the operations of MoS_2_ FETs is still under investigation due to the complex interplay of multiple mechanisms [21]. Developing a comprehensive understanding of the underlying factors is therefore crucial to accurately determine and control their influence over hysteresis [21]. The origin of hysteresis in MoS_2_ FETs has been studied by several research groups, mostly reported as arising from various intrinsic and extrinsic factors [7,16,17,22]. Primarily, charge trapping at the channel–dielectric interface or adsorption/desorption of H_2_O or O_2_ molecules have been identified as the dominant sources of hysteresis [3,16,20,21,23]. However, in MoS_2_ FETs with different channel thicknesses, the nature and density of defects contributing to hysteresis remain a question of interest requiring further investigation.

This study involves experimental investigations on the hysteresis behavior of flake-based and thin-film-based back-gate MoS_2_ FETs on a SiO_2_/Si substrate with a few-layer (FL) and thin-film (TF) channel thickness, respectively. The findings revealed that both the FL and TF MoS_2_ FETs exhibited a clockwise hysteresis behavior dominated by intrinsic and interface defects within the device channel. Notably, the TF MoS_2_ FET exhibited increased hysteresis compared to the FL FET, particularly with a higher gate voltage and sweeping range. The increased hysteresis in the TF MoS_2_ FET cannot be attributed solely to defects near the channel–dielectric interface, indicating a significant contribution from increased intrinsic defect density within the channel as the thickness increases.

## 2. Materials and Methods

### 2.1. Material Characterization

**Figure 1:**
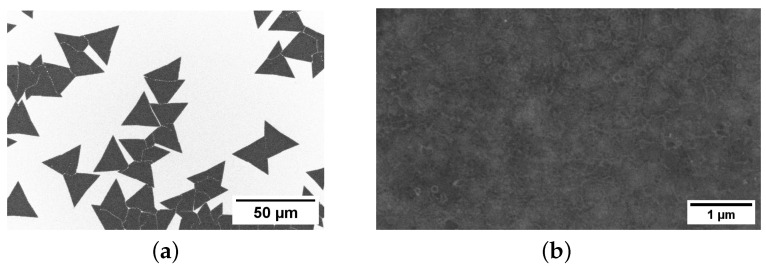
SEM images of CVD-deposited (**a**) individual MoS_2_ flakes and (**b**) a uniformly deposited thin film on a SiO_2_/Si substrate, which were employed in the fabrication of back-gate FETs.

MoS_2_ flakes and thin films were deposited through chemical vapor deposition (CVD) on a heavily doped p^+^Si substrate (orientation <100> ±0.50°, resistivity 1–5 Ωcm) covered with a 90 nm thick layer of thermally grown SiO_2_. The CVD process involved vapor-phase reactions between MoO_3_ (0.3 mg, 99% Sigma-Aldrich) and S powder (0.6 mg, 99% Sigma-Aldrich) as precursor materials, depositing MoS_2_ directly onto the SiO_2_/Si substrates within a single-zone quartz tube furnace. The reaction chember was ramped to ~750 °C and purged in a N_2_ (1000 sccm) environment for one hour before initiating the process. Throughout the deposition period, the precursor temperatures for MoO_3_ and S were maintained at ~750 °C and ~240 °C, respectively, with continuous monitoring using thermocouples. The scanning electron microscopy (SEM, Raith-150) image in Figure 1a illustrates individual MoS_2_ flakes, while Figure 1b displays a uniformly deposited thin film of MoS_2_ covering the SiO_2_/p^+^Si substrate.

**Figure 2:**
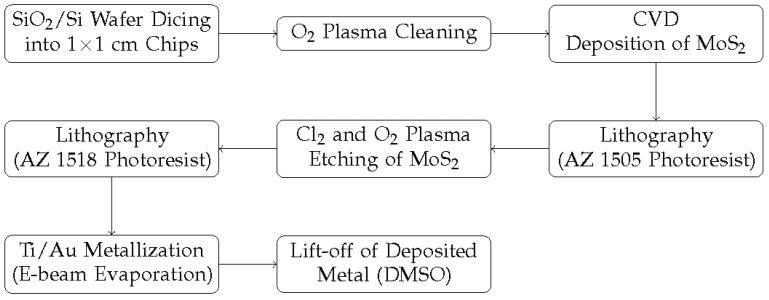
Schematic representation outlining the fabrication process steps for FL and TF MoS_2_ FETs.

**Figure 3:**
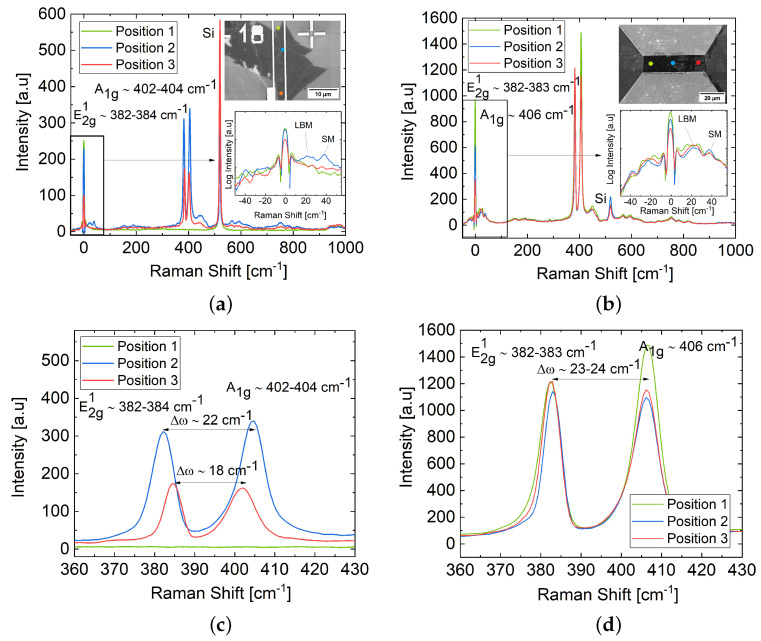
Raman spectra of MoS_2_ channel obtained from (**a**,**c**) flake-based few-layer (FL) and (**b**,**d**) thin-film-based (TF) FETs. Three distinct positions on the device channel were measured and highlighted in specific colors, illustrating unique vibrational modes of MoS_2_, including $E_{2g}^{1}$ and $A_{1g}$. Insets show the SEM image of the MoS_2_ FETs and the low-frequency (<50 cm^−1^) modes of the Raman spectrum on a logarithmic scale.

The CVD-deposited MoS_2_ flakes and thin films were subsequently characterized by Raman spectroscopy (Witec Alpha 300R, excitation wavelength of 488 nm) at room temperature. Afterward, the MoS_2_ flakes and thin-film samples were cleaned, pre-baked, coated with an adhesion promoter (HMDS), and then spin-coated at 4000 rpm with a positive resist (AZ 1505). Following this, the MoS_2_ channel was defined by patterning the resist through image inversion using a maskless lithography machine (MLA 150 Heidelberg Instruments). Exposed patterns were then developed in a developer solution (AZ 351-B) for 30 s. Subsequent to this step, the samples underwent etching using CL_2_ and O_2_ plasma for 1 min, and the resist was removed using acetone afterward. To fabricate contact leads for the back-gate FETs, both samples underwent a coating of HMDS and were subsequently spin-coated with another positive resist (AZ 1518) at 4000 rpm. The metal contacts were then defined by patterning the resist using image inversion with the maskless lithography machine. Exposed patterns were developed in a developer solution (AZ 351-B) for 30 s. Following development, the samples were metalized through electron beam evaporation to deposit the Ti (10 nm)/Au (80 nm) films. The standard lift-off process was employed later to remove the metal residuals in DMSO for 30 min, completing the contact fabrication for the back-gate MoS_2_ FETs. A simplified schematic outlining the fabrication process steps for the MoS_2_ FETs is illustrated in Figure 2. The FETs derived from individual flakes had a channel length (L) = 3.5 μm and width (W) = 15.5 μm, while those from the thin film had L = 13.5 μm and W = 50.4 μm, as shown in the SEM images of the Figure 3a,b inset. For detailed information on the deposition and device processing parameters, please refer to the reported work of Mathew, S. et al. [14,24].

The Raman spectra obtained from the channel layer of the experimental back-gate FETs based on a MoS_2_ flake and thin film are presented in Figure 3a,b, revealing two pronounced first-order Raman active modes of MoS_2_: $E_{2g}^{1}$ and A_1*g*_, corresponding to the in-plane vibration of the Mo–S bond along the base plane of the MoS_2_ sheets, and the out-of-plane vibration of S along the vertical plane of MoS_2_ [4,25,26,27]. The observed frequency difference (Δω) between the $E_{2g}^{1}$ and A_1*g*_ Raman modes at different positions, as well as the intensity ratios of Si to $E_{2g}^{1}$ (I_Si_/$I_{E_{2g}^{1}}$) and A_1*g*_ (I_Si_/$I_{A_{1g}}$), provided insights into the thickness, surface morphology, and coverage of the MoS_2_ channels [25,26,27]. The flake-based MoS_2_ FET channel layer exhibited Δω∼18–22 cm^−1^ and intensity ratios of I_Si_/$I_{E_{2g}^{1}}$ and I_Si_/$I_{A_{1g}}$ averaging ∼2 at different positions (Figure 3c), suggesting variations in surface morphology along the channel and thickness ranging from mono- to few-layer [25,26,27]. Moreover, the absence of the characteristic peaks of MoS_2_ in Figure 3a,c at position 1 along the channel layer implies that the flake structure did not fully cover the channel area uniformly. In contrast, the thin-film-based MoS_2_ FET exhibited complete coverage of the channel area and thickness variations from few-layer to multi-layers, with Δω∼23–24 cm^−1^ (Figure 3b,d), and intensity ratios of I_Si_/$I_{E_{2g}^{1}}$ and I_Si_/$I_{A_{1g}}$ averaging ∼0.15 [25,26,27].

Furthermore, the shear modes (SMs) and layer-breathing modes (LBMs) identified in the low-frequency region (<50 cm^−1^) of the Raman spectrum were scrutinized and are illustrated in the insets of Figure 3a,b on a logarithmic scale. Analyzing the low-frequency SMs and LBMs allowed for a comprehensive characterization of mono-, few-layered, and thin-film MoS_2_, complementing the high-frequency Raman measurements, as these Raman active modes also exhibit positional shifts with the layer numbers [28]. Significantly, SMs and LBMs are absent in mono-layers but display a characteristic blue (stiffening) and red (softening) shift, respectively, with the increasing number of layers from 2L to 5L [28]. However, the peak intensity and shift declines as the film thickness progresses from 5L to the bulk [28]. The low-frequency Raman peak positions of the MoS_2_ flake, presented in the inset in Figure 3a, demonstrated a close agreement with high-frequency measurements and indicated variations in thickness from monolayer to few layers. In positions 1 and 3, there were no explicit SMs and LBMs; however, they were evident in position 2. Conversely, the peak positions of MoS_2_ thin film, shown in the Figure 3b inset, indicated thin-film characteristics with minor variations in SM and LBM positions and peak intensities.

Subsequently, the surface topography of the MoS_2_ FET channels was characterized using atomic force microscopy (AFM) measurements, revealing a thickness of approximately a few layers (FL) (∼2.8 nm, ∼4 monolayers) for the flake-based MoS_2_ FET and thin film (TF) characteristics (∼34 nm) for the thin-film-based MoS_2_ FET.

### 2.2. Electrical Characterization

The electrical characterization of the FL and TF MoS_2_ FETs was conducted in a shielded probe station connected to a Keithley-4200 semiconductor characterization system at room temperature, under a N_2_ atmosphere, and in darkness, unless stated otherwise. Measurements were conducted in a two-probe configuration, with the FET contact leads serving as the source and drain, while the heavily doped p^+^Si substrate acted as the global back-gate electrode. Quantitative comparison between devices with varying dimensions (*L* and *W*) was performed by normalizing the measured current ($I_{ds(m)}$) using Equation (1).

(1) $$I_{ds}=\frac{I_{ds(m)}}{W/L}$$

**Figure 4:**
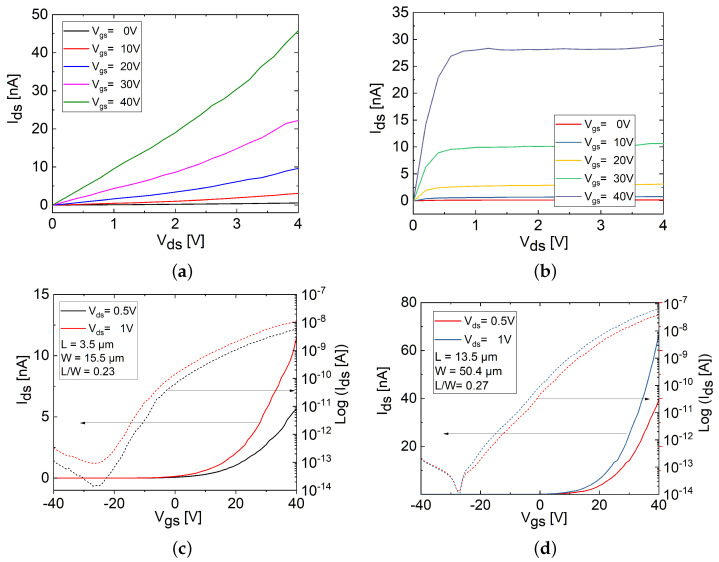
(**a**,**b**) Output ($I_{ds}-V_{ds}$) characteristics in linear scales and (**c**,**d**) transfer ($I_{ds}-V_{gs}$) characteristics plotted on linear (left *y*-axis, solid lines) and logarithmic (right *y*-axis, dashed lines) scales for FL (left column) and TF (right column) MoS_2_ FETs.

The output ($I_{ds}-V_{ds}$) characteristics of the FL and TF MoS_2_ FETs are illustrated in Figure 4a,b, respectively. Sweeping drain-source voltage ($V_{ds}$) from 0 to 4 V and varying gate-source voltage ($V_{gs}$) in steps of 10 V (0, 10, 20, 30, and 40 V) revealed the modulation of $I_{ds}$ with increasing $V_{gs}$, confirming the channel resistance was gate modulated in both FET types [3,15]. The FL MoS_2_ FET with nearly Ohmic contact properties was observed in the lower $V_{ds}$ region, whereas the TF MoS_2_ FET displayed Ohmic contact properties. Furthermore, the TF FET, as opposed to the FL FET, exhibited current saturation as $V_{ds}$ increased.

Figure 4c,d display the transfer ($I_{ds}-V_{gs}$) characteristics of the FL and TF MoS_2_ FETs plotted on linear (left *y*-axis, solid lines) and logarithmic (right *y*-axis, dashed lines) scales, which comprehensively depict the effect of the gate. The measurements illustrate the dependence of $I_{ds}$ on varying $V_{gs}$ from −40 to 40 V, while $V_{ds}$ was set to 0.5 and 1 V. Both the FL and TF FETs exhibited *n*-type behavior in the enhancement mode (normally off transistor), characterized by positive threshold voltages ($V_{th}$) of 12 and 22 V, with $I_{ds}$ of 60 pF and 47 pF at $V_{gs}$ = 0 V, respectively. Such *n*-type behavior is mostly attributed in the literature to the Fermi level pinning close to the MoS_2_ conduction band that typically results in a small but not negligible Schottky barrier height (SBH) for electron injection and a high SBH for hole injection [3,15]. Several performance parameters were extracted from the transfer characteristics of the FETs at $V_{ds}$ = 0.5 V, summarized in Table 1.

The differing performance between the FL and TF MoS_2_ FETs can be ascribed to various factors. The nearly Ohmic contact properties observed in the output characteristics of the FL FET, as opposed to the TF FET that demonstrated Ohmic contact properties, signify the presence of a higher SBH and contact resistance ($R_{c}$) in the FL FET. The experimental investigation of the FL FET revealed that the FL channel contains abundant grain boundaries and variations in morphology. Due to its low-dimensional nature, defects within the FL MoS_2_ channel at the MoS_2_/SiO_2_ interface and impurities on the substrate surface adversely affected the device performance and contributed to an increased channel resistance ($R_{ch}$) [3,15,18,29,30]. Consequently, the total resistance ($R_{t}$) of the FL FET (∼ 25 mΩ/μm) was found to be higher than that of the TF FET (∼ 12 mΩ/μm), extracted from the output characteristics at $V_{ds}=4\;V$ and $V_{gs}=40\;V$.

In contrast, the Ohmic contact properties, lower $R_{t}$, current saturation, and improved electron transport observed in the TF FET are attributed in the literature to a higher density of states (DoS) and lower SBH, facilitated by several other factors [18,29]. One crucial factor is the diminished influence of interface trap states facilitated by the relatively long distance between the upper layers of the TF structure and the substrate [18]. Additionally, the presence of underlying layers in the TF act as a screening effect, further minimizing the impact of interface impurities and surface scattering on the TF FET performance [18]. Moreover, the upper layers of the TF channel exhibit a relatively low density of localized trap states compared to the FL channel, thereby facilitating improved electron transport [18,29]. Furthermore, the TF FET demonstrates the capability to generate higher drive currents through multiple conducting channel layers, which collectively contributed to the significant improvements in the maximum $I_{on}$, $I_{on}/I_{off}$, and $\mu_{fe}$ by over one order of magnitude compared to the FL FET, as summarized in Table 1 [18,22,29,31].

Nevertheless, increasing the number of layers in the TF MoS_2_ structure also gave rise to an increase in the intrinsic defect density, originating from structural inhomogeneity, vacancies, dislocations, or grain boundaries within the channel material [13,15,18]. This increase, in turn, resulted in a higher SS value in the TF FET due to the emergence of defect states within the MoS_2_ channel volume, coupled with the lower electrostatic gate control compared to the FL FET [13,18].

## 3. Results and Discussion

**Figure 5:**
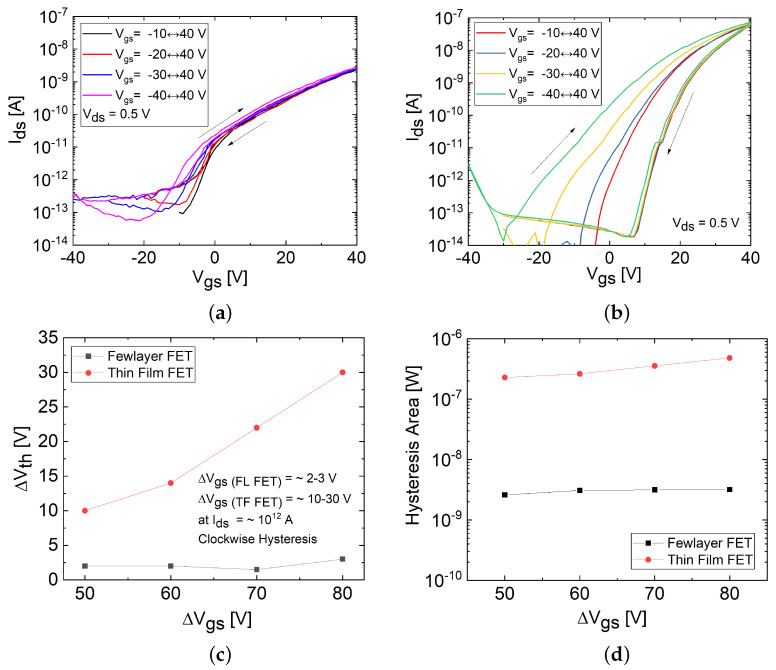
Double-sweep transfer characteristics of (**a**) FL and (**b**) TF MoS_2_ FETs with a decreasing negative start cycle at $V_{ds}$ = 0.5 V, displaying clockwise hysteresis (+$\Delta V_{th}$) with arrows indicating the direction, and an asymmetry in $V_{th}$ shift in the forward and backward sweep curves found to be common for both device types. (**c**) Comparison of the $\Delta V_{th}$ increase and (**d**) the hysteresis areas (*H*) with increasing $V_{gs}$ sweep ranges ($\Delta V_{gs}$) between FL and TF FETs.

Fluctuations in the number of charge carriers observed during forward (off state to on state) and backward (on state to off state) voltage sweeps in the FL and TF MoS_2_ FETs, shown in Figure 5a,b, indicate the presence of trap states capturing and releasing charges [3,21]. Trap states originating from several intrinsic and extrinsic factors can affect the charge carrier density, channel current, and lead to $V_{th}$ shifts ($\Delta V_{th}$) as well as hysteresis [3,7,16,17,21,22]. Intrinsic factors generating charge trap states include defect states inherent to the MoS_2_ channel (i.e., dislocations, grain boundaries, Mo or S vacancies, etc.), the presence of mobile ions (i.e., Na^+^ and K^+^) in the dielectric SiO_2_ layer, and oxide traps close to the MoS_2_/SiO_2_ interface, or at the SiO_2_/p + Si interface [3,7,17,21,22,32]. Extrinsic factors involve charge-trapping states induced by external sources, including adsorbed moisture (i.e., O_2_ or H_2_O molecules) or process residues deposited on the MoS_2_ channel surface or at the MoS_2_/SiO_2_ interface [3,7,16,17,21]. Environmental conditions including pressure, temperature, humidity, and the photosensitivity of MoS_2_ can also have a significant impact on hysteresis [3,7,16,17,21,33]. However, identifying the specific factors responsible for hysteresis is challenging due to the complex interplay of multiple mechanisms, which can be analyzed through temperature-dependent hysteresis mechanisms, as well as the nature of hysteresis, whether clockwise or counterclockwise [21,22,32,33]. Clockwise hysteresis is associated with the intrinsic and interface traps within the channel material, adsorbates on the channel surface, or oxide traps near the channel–dielectric interface [21,32]. In contrast, counterclockwise hysteresis is induced by mobile ions in the dielectric layer at room temperature or oxide traps near the dielectric–gate substrate interface at high temperatures [21].

**Figure 6:**
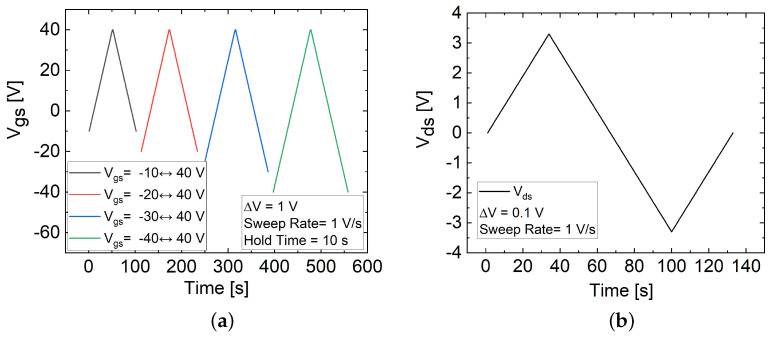
Illustration of double-sweep curves for the MoS_2_ FETs: (**a**) $I_{ds}-V_{gs}$ curve with a decreasing negative $V_{gs}$ value at the beginning of a cycle, and (**b**) $I_{ds}-V_{ds}$ curve with varying $V_{ds}$ throughout a cycle.

In this study, the underlying causes of hysteresis were investigated by measuring the hysteresis dependence on varying forward and backward $V_{gs}$ sweep conditions conducted on both FET types. As illustrated in Figure 6a, $V_{gs}$ was initially swept from a negative bias to a positive maximum bias (−10 to 40 V), and then reversed back to the initial negative bias value (40 to −10 V). Each sweep cycle involved gradually decreasing the minimum $V_{gs}$ start value (−10, −20, −30, and −40 V) while keeping the maximum $V_{gs}$ value (40 V) constant. This sequence of sweeps, referred to as the decreasing negative start cycle, allowed for an exploration of the impact of varying the trap state occupancy on hysteresis characteristics, including the clockwise or counterclockwise orientation of hysteresis, represented by the (+) or (−) magnitude of $\Delta V_{th}$.

Figure 5a,b present the double-sweep transfer characteristics of the FL and TF FETs, measured in a decreasing negative start cycle, with $V_{ds}$ set to 0.5 V. Both figures demonstrate that the channel current ($I_{ds}$) during the forward sweep consistently exceeded that during the backward sweep in both device types, indicating clockwise hysteresis (+$\Delta V_{th}$). Moreover, the magnitude of hysteresis also increased with the increasing $V_{gs}$ sweep range ($\Delta V_{gs}$), exhibiting an asymmetry in the $V_{th}$ shift observed between the forward and backward sweep curves during the double-sweep cycles [3,20,23]. Specifically, with the gradual decrease in the starting $V_{gs}$ value at the onset of each cycle, a substantial $V_{th}$ shift in the forward sweep curves, but a minor $V_{th}$ shift in the backward sweep curves, were found to be common for both device types [3,20,23]. For a quantitative comparison of the hysteresis effect between the FL and TF FETs, the changes in $\Delta V_{th}$ and the hysteresis area (*H*) with increasing $\Delta V_{gs}$ were extracted from the double-sweep transfer curves depicted in Figure 5a,b. Equation (2) was used to obtain *H*, defined as the area between the forward and backward $I_{ds}$ curves of the double-sweep transfer characteristics. The results presented in Figure 5c,d indicate that as $\Delta V_{gs}$ increased, both $\Delta V_{th}$ and *H* exhibited an increase, with the TF FET showing a stronger increase in $\Delta V_{th}$ and *H* compared to the FL FET. The FL FET exhibited a $\Delta V_{th}$ of 2–3 V, whereas the TF FET showed a $\Delta V_{th}$ ranging from 10 to 30 V with increasing $\Delta V_{gs}$, as illustrated in Figure 5c. Figure 5d demonstrates that the TF FET exhibited a notably strong increase in *H* as $\Delta V_{gs}$ increased, approximately two orders of magnitude higher than that of the FL FET.

(2) $$H=\int_{V_{\min}}^{V_{\max}}I_{ds}\left(V_{gs}\right)\;dV_{gs\;\left(\mathrm{forward}\right)}-\int_{V_{\min}}^{V_{\max}}I_{ds}\left(V_{gs}\right)\;dV_{gs\;\left(\mathrm{backward}\right)}$$

Studies conducted on explaining the occurrence of clockwise hysteresis and the asymmetry in $V_{th}$ shift between the forward- and backward-sweep curves in the transfer characteristics have emphasized that the observed hysteresis in MoS_2_ FETs primarily originates from structural defects, such as grain boundaries or sulfur vacancies (SVs), in the MoS_2_ channel and at the MoS_2_/SiO_2_ interface [3,17,20,23,30,32]. These structural defects introduce localized donor-like charge trapping states within the energy band gap of MoS_2_, located below the Fermi level at the semiconductor–dielectric interface and within the bulk [3,17,23,30]. The donor-like trap states are neutral when occupied by electrons, and positively charged when unoccupied [3,17,23]. The forward $V_{gs}$ sweep facilitates the trapping of electrons from the channel into these donor-like trap states, neutralizing them as they become filled [3,17,22,23,32]. This results in a decrease in the carrier density, channel current, and an increase in the $V_{th}$ during the following backward sweep [3,17,22,23,32]. Conversely, the backward sweep facilitates charge de-trapping, where these trap states release electrons back into the channel, leaving the traps empty again with positive charges [3,17,22,23,32]. This leads to an increase in the carrier density, channel current, and a decreased $V_{th}$, as observed during the following forward sweep [3,17,22,23,32]. As a consequence, distinct levels of drain current ($I_{ds}$), a positive shift in the $V_{th}$, as well as clockwise hysteresis (+$\Delta V_{th}$) were observed [3,17,23,32]. Furthermore, since the positively charged donor-like traps are initially unoccupied with electrons at the beginning of a forward sweep, the maximum value of positive charges will be imposed on the gate. As the forward sweep progresses, most of the trap states become neutralized by electrons. Consequently, at the beginning of the backward sweep, the neutralized traps exert minimal influence on the gate. This dual effect, consisting of the maximum effects of positive charges during the forward sweep followed by the minimal effects of neutralized states during the backward sweep affecting the gate control, contributes to an asymmetry in the shifting of $V_{th}$. This asymmetry manifests as a rapid shift in $V_{th}$ during the forward sweep and a slight shift during the backward sweep [23]. In this study, investigating the influence of varying trap-state occupancy on hysteresis characteristics involved reducing the negative starting $V_{gs}$ in each cycle at the onset of the forward sweep, illustrated in Figure 6a, which exposed more donor-like positively charged traps at the interface. This led to a shift of $V_{th}$ toward the negative direction due to the increased positively charged states imposed on the gate, resulting in increased hysteresis, as indicated by the changes in $\Delta V_{th}$ and *H* depicted in Figure 5c,d, particularly with a larger $V_{gs}$ sweep range ($\Delta V_{gs}$) [3,20,23].

**Figure 7:**
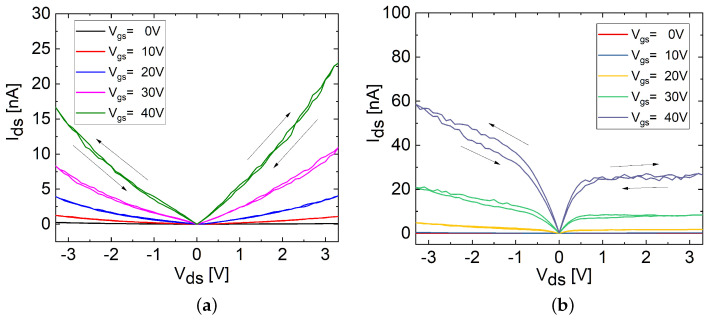
Double-sweep output characteristics of (**a**) FL and (**b**) TF MoS_2_ FETs with increasing $V_{gs}$ conditions in linear scale, with clockwise hysteresis indicated by the arrows.

Afterward, the hysteresis characteristics of both the FL and TF FETs were investigated in the double-sweep output curves. As illustrated in Figure 6b, the $V_{ds}$ sweep started from 0 V and moved in the positive direction (0 to 3.3 V), then reversed towards negative values (−3.3 to 3.3 V) and finally returned to the initial state (3.3 to 0 V). In each sweep cycle, $V_{gs}$ was incremented from 0 to 40 V in steps of 10 V (0, 10, 20, 30, and 40 V). The output curves of the FL and TF FETs, presented in Figure 7a,b, exhibited a consistent increase in clockwise hysteresis with increasing $I_{ds}$. This behavior was strongly influenced by the modulation of $V_{gs}$, affecting the carrier density in the channel, leading to the screening effect of Coulomb scatterers and changes in the charge distribution as well as the interaction with traps, particularly due to the filling of traps near the transport band edge [3,20,32]. According to the literature, the higher hysteresis and increased $I_{ds}$ of the TF FETs are related to the higher density of states (DoS) reported for the TF FETs compared to the FL FETs [29].

The clockwise hysteresis in both device types at room temperature suggests that the underlying sources of hysteresis are similar, and negates the idea that hysteresis was caused by the presence of ions (Na^+^ and K^+^) in the SiO_2_ layer or by oxide traps near the p^+^Si gate. Because the former factor would lead to counterclockwise hysteresis, and the latter does not exhibit hysteresis at room temperature [21,32]. However, reported studies indicate that in addition to intrinsic or interface defects of MoS_2_, the adsorption of O_2_ or H_2_O molecules on the MoS_2_ channel surface or at the MoS_2_/SiO_2_ interface could also contribute to clockwise hysteresis in an ambient environment [3,20,21,32]. Nevertheless, the results obtained in our study do not support the perception that adsorbate-mediated hysteresis played a dominant role in the observed clockwise hysteresis. This is because our experimental setup was meticulously designed to mitigate changes in humidity. The introduction of N_2_ purging into the probe station housing played a crucial role in maintaining a controlled environment during the electrical characterization of the FL and TF MoS_2_ FETs. Prior to introducing the device under test (DUT) into the shielded probe station, the measurement chamber underwent extensive N_2_ purging for several hours. Additionally, as a precautionary measure, before conducting measurements, the devices were kept inside the chamber for several hours to minimize the potential moisture accumulation on the top surface. The DUT was enclosed within the closed measurement chamber throughout the measurement process, minimizing the influence of gas adsorption [32].

**Figure 8:**
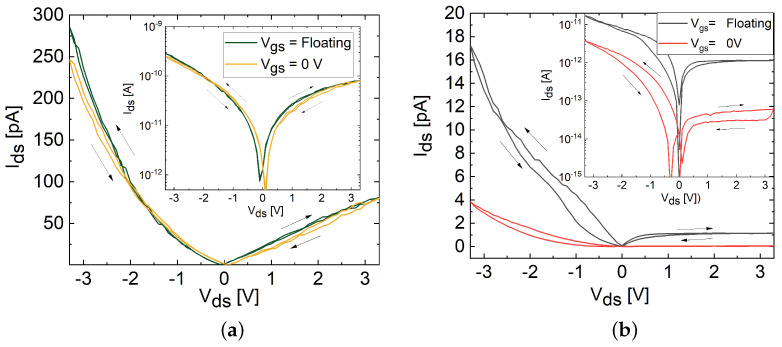
Double-sweep output characteristics of (**a**) FL and (**b**) TF MoS_2_ FETs at floating and grounded ($V_{gs}$ = 0 V) gate conditions in linear scales, with arrows indicating the direction of the clockwise hysteresis loop. Insets represent the measurements on a logarithmic scale.

Subsequently, an output measurement analysis involving both floating and grounded ($V_{gs}$= 0 V) gate conditions was conducted on both devices to analyze the impact of intrinsic and interface defects within the MoS_2_ channel on hysteresis characteristics, depicted in Figure 8a,b. The floating-gate measurement involved sweeping $V_{ds}$ without applying $V_{gs}$. The $V_{ds}$ sweep was varied from 0 V to 3.3 V, then from 3.3 V to −3.3 V, and finally back to 0 V, as illustrated in Figure 6b. The results demonstrate that the TF FET exhibited increased hysteresis compared to the FL FET even without the application of $V_{gs}$. Furthermore, when the gate was grounded ($V_{gs}$ = 0 V), an increase in hysteresis was observed in both devices, accompanied by a decrease in the channel current ($I_{ds}$).

This disparity in hysteresis under the floating and grounded gate conditions suggests that under the floating-gate configuration, charge carriers likely traversed through the bulk of the channel, taking advantage of the path with a lower defect density and lower resistance, rather than primarily passing near the interface [18]. Consequently, the channel current was higher where the observed hysteresis can be attributed to the presence of intrinsic defects within the MoS_2_ channel volume, which tends to increase with layer thickness [18]. This observation aligns with the higher SS exhibited by the TF FET compared to the FL FET, indicating a higher defect density within the bulk of the TF channel. However, when the charge carriers were transported through the MoS_2_/SiO_2_ interface under the grounded ($V_{gs}$ = 0 V) condition, they encountered interface defects and impurities at the substrate surface. This interaction resulted in a decreased current compared to the floating-gate configuration [7,18]. The increased hysteresis observed in the output curves can be due to the enhanced trapping of charge carriers at the interface rather than in the bulk.

**Figure 9:**
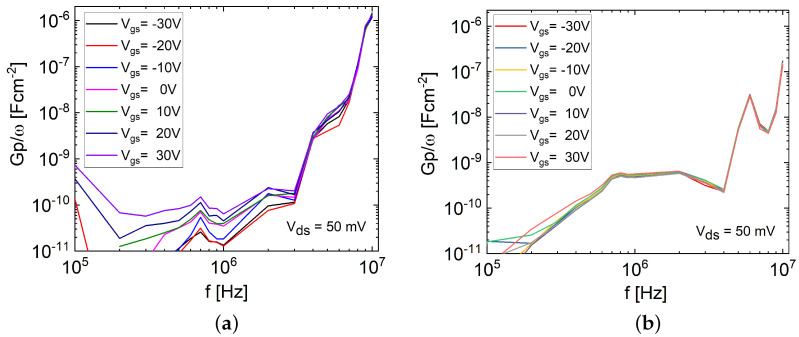
Conductance–frequency ($G_{p}/\omega -f$) measurements of (**a**) FL and (**b**) TF MoS_2_ FETs. Measurements involve sweeping $V_{gs}$ from depletion to accumulation region (−30 to 30 V) within the frequency range of 100 kHz to 10 MHz.

To quantitatively evaluate the density of the interface and intrinsic bulk trap states in the FETs and assess their impact on device performance, conductance–frequency ($G_{p}/\omega -f$) measurements were performed [34]. Throughout the measurement process, the source and drain electrodes were shorted and connected to the low terminal of the capacitance measurement unit (CMU), while the back-gate electrode was connected to the high terminal. The bias on the high terminal was adjusted accordingly. The $G_{p}/\omega -f$ measurement involved sweeping the $V_{gs}$ from the depletion to the accumulation region (−30 to 30 V) within the frequency range of 100 kHz to 10 MHz, as shown in Figure 9a,b. A conductance analysis was performed with an AC signal amplitude of 50 mV to avoid false conductance values arising from the harmonics in the signal frequency [34]. During the measurement, the CMU measured the parallel capacitance ($C_{m}$) and conductance ($G_{m}$).

Based on the measured parallel $C_{m}-G_{m}$ combination at $V_{gs}$ = 30 V, the normalized equivalent parallel conductance ($G_{p}/\omega$) and the density of traps at the interface ($D_{t(i)}$) were determined using Equations (3) and (4), respectively [34,35,36].

(3) $$\frac{G_{p}}{\omega }=\frac{\omega G_{m}C_{ox}^{2}}{G_{m}^{2}+\omega^{2}{\left(C_{ox}-C_{m}\right)}^{2}}$$

(4) $$D_{t(i)}=\frac{2.5}{q}\;\cdot \;\frac{G_{p}}{\omega }$$

Here, ω represents the angular frequency, and the magnitude of the $G_{p}/\omega$ peak corresponds to the maximum $D_{t(i)}$ [34,35,36]. The analysis revealed that the FL MoS_2_ FET exhibited a higher $D_{t(i)}$ value of $2.3\times 10^{13}$ states/cm^2^-eV, whereas the TF MoS_2_ FET had a lower value of $2.7\times 10^{12}$ states/cm^2^-eV. Furthermore, the sub-threshold swing (SS) method was employed to estimate the density of the traps inside the bulk ($D_{t(b)}$) of the MoS_2_ channel. The extracted SS values for the FL and TF FETs were utilized to obtain the channel capacitance ($C_{ch}$) using Equation (5) [22,35]. Subsequently, using the Equation (6), which warranted the careful consideration of both the geometric capacitance and trapping effects, the total density of the traps within the MoS_2_ channel volume ($D_{t(ch)}=D_{t(b)}+D_{t(i)}$) was determined [35].

(5) $$SS=\frac{ln10K_{B}T}{q}\left[1+\frac{C_{ch}}{C_{ox}}\right]=\frac{ln10K_{B}T}{q}\left[1+\frac{C_{s}+C_{it}}{C_{ox}}\right]$$

(6) $$C_{ch}=C_{geometry}+C_{traps}=\frac{\epsilon_{0}\epsilon_{r}\;\cdot \;A}{d}+qD_{t(ch)}$$

Here, $K_{B}T/q,C_{ch}\left(C_{ch}=C_{s}+C_{it}\right),C_{s},C_{it},C_{ox},q,\epsilon_{0},\epsilon_{r},A,$ and *d* represent the thermal voltage, channel capacitance, semiconductor capacitance, interface capacitance, oxide (SiO_2_) capacitance, elementary charge, vacuum permittivity, relative (MoS_2_) permittivity, semiconductor channel area, and thickness, respectively. Approximating $D_{t(b)}$ from extracted $D_{t(ch)}$ and $D_{t(i)}$, the contribution of both $D_{t(i)}$ and $D_{t(b)}$ to the observed hysteresis in the FL and TF devices was determined. The summarized results are presented in Table 2, indicating that the high $D_{t(i)}$ played a significant role in causing hysteresis in the transport properties of both investigated device types. However, in the case of the TF FET, the increased hysteresis compared to the FL FET cannot be fully explained by $D_{t(i)}$ alone, despite having a decreased value. The elevated hysteresis observed in the TF MoS_2_ FET was found to be attributed to the TF MoS_2_ channel exhibiting an order of magnitude higher $D_{t(b)}$, contributing along with the $D_{t(i)}$. Therefore, when analyzing hysteresis behavior in 2D FETs, particularly in TF FETs, the impact of bulk defects, along with the presence of interface traps and adsorbates on the unpassivated channel surface during fabrication, should be considered [20].

## 4. Conclusions

In conclusion, the experimental investigations on both the FL and TF MoS_2_ FETs revealed gate-modulated clockwise hysteresis behavior and an asymmetry in the $V_{th}$ shift in the forward- and backward-sweep curves in the double-sweep transfer characteristics. The magnitude of hysteresis ($\Delta V_{th}$ and *H*) was found to increase with higher $V_{gs}$ and larger voltage sweep ranges, with the TF FET exhibiting a stronger increase in $\Delta V_{th}$ and *H* compared to the FL FET. The observed clockwise hysteresis in both devices indicated similar underlying sources of hysteresis, primarily attributed to the intrinsic and interface trap states in the MoS_2_ channel and at the MoS_2_/SiO_2_ interface. The asymmetry in $V_{th}$ shifting was ascribed to a dual effect involving the impact of positive charges during the forward sweep, followed by the minimal effect of neutralized states during the backward sweep, influencing the gate control.

Further investigation of double-sweep output characteristics under both floating and grounded gate configurations inferred that under floating-gate conditions, hysteresis was predominantly influenced by the intrinsic defects within the bulk of the channel, leading to higher hysteresis in the TF FETs due to increased defect density with layer thickness. Conversely, under grounded-gate conditions, interface defects played a more significant role, resulting in decreased channel current ($I_{ds}$) and increased hysteresis compared to the floating-gate configuration.

The conductance–frequency ($G_{p}/\omega -f$) measurements determined that the FL FET possessed a greater density of interface traps ($D_{t(i)}$) compared to the TF FET. Despite the roughly similar order of magnitude in the channel thickness between the FL (∼2.8 nm) and TF FETs (∼34 nm), the bulk trap density also played a proportional role in the thickness. In TF FET, there was an order of magnitude higher density of intrinsic traps ($D_{t(b)}$) within the MoS_2_ channel bulk, contributing to the increased hysteresis along with the $D_{t(i)}$. Consequently, the elevated hysteresis observed in the TF MoS_2_ devices, as opposed to the FL devices, was primarily attributed to the significant contribution from intrinsic defects within the channel volume rather than interface defects. This underscores the importance of considering both the intrinsic defects within the bulk and the interface defects of the channel layer when analyzing hysteresis in MoS_2_ FETs, especially in TF FETs. The choice between FL and TF MoS_2_ devices depends on the specific requirements for memristive applications, such as hysteresis tolerance and scaling capabilities.

## Figures and Tables

**Table 1:** Performance parameters for FL and TF FETs at $V_{ds}=0.5$ V.

Parameter	Units	FL-FET	TF-FET
$I_{on}$ ($V_{gs}$ = 40 V)	nA	∼6	∼40
$I_{off}$ ($V_{gs}$ = −28 V)	fA	∼18	∼15
$I_{on}/I_{off}$	-	∼$10^{5}$	∼$10^{6}$
$V_{th}$ (Linear Extrapolation)	V	12	22
$\mu_{fe}$	cm^2^/Vs	0.02	0.17

**Table 2:** Comparison of parameters between FL and TF MoS_2_ FETs.

Parameters	FL MoS_2_ FET	TF MoS_2_ FET
$D_{t(ch)}$ [states/cm^2^-eV]	$2.5\times 10^{13}$	$3.8\times 10^{13}$
$D_{t(i)}$ [states/cm^2^-eV]	$2.3\times 10^{13}$	$2.7\times 10^{12}$
$D_{t(b)}$ [states/cm^2^-eV]	∼$10^{12}$	∼$10^{13}$

FET parameters, including the density of states inside the MoS_2_ channel ($D_{t(ch)}$), which comprises the density of states at the MoS_2_/SiO_2_ interface ($D_{t(i)}$) and within the channel bulk ($D_{t(b)}$), for both FL and TF MoS_2_ devices.

## Data Availability

The data presented in this study are available on request from the corresponding authors.
